# Predictors of the observed high prevalence of loss to follow-up in ART-experienced adult PLHIV: a retrospective longitudinal cohort study in the Tanga Region, Tanzania

**DOI:** 10.1186/s12879-023-08063-9

**Published:** 2023-02-14

**Authors:** Stella E. Mushy, Expeditho Mtisi, Eric Mboggo, Simon Mkawe, Khadija I. Yahya-Malima, John Ndega, Frida Ngalesoni, Aisa Muya

**Affiliations:** 1 Department of Community Health Nursing, School of Nursing, Muhimbili University of Health and Allied Sciences, Dar es Salaam, Tanzania; 2 Department of General Studies, Dar Es Salaam Institute of Technology, Dar es Salaam, Tanzania; 3 Department of Nursing Management, School of Nursing, Muhimbili University of Health and Allied Sciences, Dar es Salaam, Tanzania; 4 Amref Health Africa, Ali Hassan Mwinyi Road, Dar es Salaam, Tanzania

**Keywords:** Adult HIV positive, Antiretroviral therapy, HIV/AIDS, Loss to follow-up, Predictors, Africa, Tanzania

## Abstract

**Background:**

Antiretroviral therapy (ART) programs have expanded rapidly, and they are now accessible free of charge, yet "loss to follow-up, LTFU" is still a national public health issue. LTFU may result in treatment failure, hospitalization, increased risk of opportunistic infections and drug-resistant strains, and shortening the quality of life. This study described the rates and predictors of LTFU among adults living with human immunodeficiency virus (PLHIV) on ART in the Tanga region, Tanzania.

**Methods:**

A retrospective longitudinal cohort study was conducted between October 2018 and December 2020 in Tanga's care and treatment health services facilities. The participants were HIV adult PLHIV aged 15 years and above on ART and attended the clinic at least once after ART initiation. LTFU was defined as not taking ART refills for 3 months or beyond from the last attendance of a refill and not yet classified as dead or transferred out. Cox proportional hazard regression models were employed to identify risk factors for LTFU. P values were two-sided, and we considered a p < 0.05 statistically significant.

**Results:**

57,173 adult PLHIV were on ART of them, 15,111 (26.43%) were LTFU, of whom 10,394 (68.78%) were females, and 4717 (31.22%) were males. Factors independently associated with LTFU involved age between 15 and 19 years (HR: 1.85, 95% CI 1.66–2.07), male sex (HR: 2.00 95% CI 1.51–2.62), divorce (HR: 1.35, 95% CI 1.24–1.48), second-line drug type (HR: 1.13, 95% CI 1.09–1.18), poor drug adherence (HR: 1.50, 95% CI 1.23–1.75), unsuppressed viral load (HR: 2.15, 95% CI 2.02–2.29), not on DTG-related drug (HR: 7.51, 95% CI 5.88–10.79), advanced HIV disease WHO stage III and IV (HR: 2.51, 95% CI 2.32–2.72). In contrast to cohabiting, ART duration < 1 year, and being pregnant showed a reduced likelihood of LTFU.

**Conclusion:**

A high prevalence of LTFU was observed in this study. Young age, not using DTG-based regimen, WHO clinical stage IV, poor drug adherence, male sex, unsuppressed viral load, divorcee, and second-line regime were independently associated with LTFU. To reduce LTFU, evidence-based interventions targeting the identified risk factors should be employed.

## Background

Globally in 2021, 38.4 million people were living with Human Immunodeficiency Virus (HIV); 36.7 million were adults aged 15 years or older; 54% were women and girls [1]. HIV/AIDS pandemic remains a serious public health concern where effective use of antiretroviral therapy (ART) has significantly reduced HIV transmission, HIV-related morbidity and mortality, and improved quality of life [2]. Globally in 2021, of the people living with HIV/AIDS, 29.1 million (76%) of all adults aged 15 years and older were accessing treatment (80% female vs. 70% male) [1]. Between 2010 and 2021, there was evidence of reduced AIDS-related mortality by 57% among women and 47% among men due to ART use [1]. In addition, the risk of HIV transmission from PLHIV who adhered well to ART with regular follow-up of their uninfected sexual partners decreased significantly by 96% [3]. However, despite the ready accessibility of ART services, disproportions of its accessibility still exist.

Loss to follow-up (LTFU) significantly impedes treatment success and improvement of HIV treatment outcomes among PLHIV on ART [2]. In Tanzania, the estimated PLHIV LTFU after enrollment in HIV care and ART programs was 31%, of whom only 8% were confirmed dead, while most of the LTFU had no known cause [4]. A study conducted in Dar es Salaam, Tanzania, found that 19.5% of the people-initiated ART were LTFU at the end of 24 months of follow-up [5]. Multiple studies in sub-Saharan Africa (SSA) found factors like age, gender, education, ART regimen, CD4 count, duration of treatment, and extremes of weight as predictors of LTFU [5–9]. In SSA, a systematic review of 39 LTFU cohorts and 22,6307 PLHIV found LTFU to be 22.6% at 12 months and 23% to 30% at 24 months [10], while a review of 180,718 PLHIV from six regions found 19.9% LTFU rate [11]. Furthermore, a study of 4206 individuals who initiated ART in a large HIV program in Nigeria indicated that 24.8% were LTFU after 10 years [12].

Mortality following LTFU in developing countries ranges from 12 to 87% [13]. Loss to follow-up will also increase the risk of drug failure, hospital admission, HIV-related morbidity, and the risk of transmitting drug-resistant strains and shortens the quality of life of PLHIV [10, 14, 15]. These adverse effects can be addressed by utilizing long-term routine collected data in our setting to describe how PLHIV, environments, and related factors affect LTFU.

The Tanzanian government targeted a 95% HIV viral load suppression rate for people on ART care and treatment to end the AIDS epidemic by 2030 [16]. Data on the rate and predictors of LTFU among adults is crucial to achieving this plan. Thus, the current study aimed to describe the rate and predictors of LTFU among adult PLHIV who are on ART in the Tanga region. The results of this study will inform policymakers, program planners, and implementers working at various levels of HIV/AIDS control programs to improve patient retention to ensure the country attains the 95–95–95 targets by 2030.

## Methods

### Study design and setting

This study was a retrospective longitudinal cohort analysis of adult PLHIV attending care and treatment clinics within eight districts supported by Amref Health Africa Tanzania in the Tanga region from October 2018 to December 2020. The HIV prevalence in Tanga is 5% (3.7 males: 6.2 females), a bit higher than the country's HIV prevalence of 4.9% (3.4% males and 6.3% females [17].

### Study population and sampling

The participants were adult PLHIV aged 15 years and above on ART and attended the clinic at least once after ART initiation. Data on PLHIV on ART from October 2018 to December 2020 was extracted from the Care and Treatment Clinics form number 2 (CTC2) database, whereby all PLHIV below 15 years of age were excluded.

### Study variables and analysis

The following variables were considered as possible determinants of loss to follow-up; sex (male, female); age (15–24; 25–34; 35–44; 45–54; 55 +); BMI (< 18.5, 18.5–< 25, 25–< 30, 30 +); WHO HIV disease stage (I, II, III, IV); district of residence (Handeni, Kilindi, Korogwe, Lushoto, Mkinga, Muheza, Pangani and Tanga); pregnant state (yes, no); marital status (Cohabiting, Divorced, Married, single and Widow); ART duration (≤ 1, > 1 years); adherence (good, poor); Viral load suppression (suppressed, non-suppressed); ART regimen (first line, second line); DTG based drug (yes, no).

### Operational definitions

This study aimed to identify factors associated with LTFU using two-year data retrieved from the CTC2 database. In this study, LTFU was defined as missing clinic visits for 90 days or more consecutively from the last clinic attendance for drug refills and not confirmed dead or transferred out [18]. Confirmed deceased PLHIV and those transferred to other health facilities not supported by Amref were censored. Confirmed Transfer out were those PLHIV who have initiated ART and later received official transfer letter to continue ART services to other non-Amref supported health facilities. Death of PLHIV was ascertained when it was documented in the ART care and treatment follow-up sheet by health care professionals. In this study, good ART adherence is considered when ≥ 95% of the medication is taken as prescribed, meaning three or fewer doses are missed. In contrast, poor adherence is considered when ≤ 85% of the medication is not taken as prescribed, i.e., nine or more doses are missed [18].

### Analysis

We did a descriptive statistical analysis to provide basic information about the variables of interest in our study to highlight potential relationships between the variables. Numbers and frequencies were used to describe categorical variables, while means/medians and interquartile ranges were used to describe continuous variables. In addition, we used bivariate analysis to ascertain the degree of association between independent and dependent variables in various periods.

We used Cox proportional hazard regression models to describe the hazard ratio of participants lost to follow-up with an associated 95% confidence interval (CI). We used Kaplan–Meier curves to estimate the cumulative incidence of loss to follow-up. The death date and last clinic visit marked the end time of follow-up. Potential risk factors that were statistically significant at a p-value of 0.2 or less in univariate analysis were included as potential confounders in multivariate models on a two-sided test with a significant p-value less than or equal to 0.05. We used the missing indicator method to handle missing data. Statistical analysis was performed using the SAS^®^ statistical software package, Release 9.3 (Cary, NC, USA).

## Results

### Study population characteristics

In the 2-year program review period, the median age of the adult PLHIV was 42.6 (interquartile range, IQR: 35–52). The median CD4 count, cell/mm^3^ 478 (IQR: 286–714). As shown in Tables 1 and 2, a total of 57,173 adults PLHIV ever on ART, females were more (69.21%), the majority (28.94%) lived in the Tanga district, and most (76.81%) had received ART for more than one year and the majority (97.89%) on first-line regimen. The majority (97.03%) had good drug adherence, and 75.08% of the participants were virally suppressed. Results showed that most (99.67%) never had a TB history, and 77.43% of the participants were on a DTG-based regimen. More than half (52.37%) of the participants were married, and only 2.19% of women were pregnant. The majority (73.5) % (n = 42,062) were retained in the study, while 26.43% (n = 15,111) were loss to follow-up.

**Table 1:** Socio-demographic factors and their association with LTFU and active status

Sample characteristics	LTFU		Total (%)
	No (%)	Yes (%)	
Median age (IQR)	42.6 (35–52)		
Age group, years			
15–24	2784 (66.36)	1411 (33.64)	4195 (7.34)
25–34	7312 (67.84)	3467 (32.16)	10,779 (18.85)
35–44	12,472 (74.11)	4356 (25.89)	16,828 (29.43)
45–54	11,542 (77.39)	3373 (22.61)	14,915 (26.09)
55 +	7952 (76.05)	2504 (23.95)	10,456 (18.29)
Sex			
Female	29,174 (73.73)	10,394 (26.27)	39,568 (69.21)
Male	12,888 (73.21)	4717 (26.79)	17,605 (30.79)
District			
Handeni	4755 (69.98)	2040 (30.02)	6795 (11.88)
Kilindi	1614 (65.32)	857 (34.68)	2471 (4.32)
Korogwe	6559 (73.62)	2350 (26.37)	8909 (15.58)
Lushoto	4457 (75.43)	1452 (24.57)	5909 (10.34)
Mkinga	2123 (76.73)	644 (23.27)	2767 (4.84)
Muheza	7397 (70.04)	3164 (29.96)	10,561 (18.47)
Pangani	2375 (73.83)	842 (26.17)	3217 (5.63)
Tanga	12,782 (77.26)	3762 (22.74)	16,544 (28.94)
Pregnant status			
No	22,765 (73.57)	8180 (26.43)	30,945 (97.81)
Yes	435 (62.86)	257 (37.14)	692 (2.19)
Marital status			
Cohabiting	589 (74.74)	198 (25.26)	787 (1.47)
Divorced	4235 (75.76)	1355 (24.24)	5590 (10.5)
Married	20,838 (74.77)	7031 (25.23)	27,869 (52.37)
Single	10,836 (70.33)	4571 (29.67)	15,407 (28.95)
Widow	2686 (75.26)	883 (24.74)	3569 (6.71)

**Table 2:** Clinical characteristics and its association with LTFU

Sample characteristic	LTFU			p-value
	No (%)	Yes (%)	Total	
Viral load suppression				
Not suppressed	2602 (68.22)	1212 (31.78)	3814 (24.92)	< 0.0001
Suppressed	9777 (85.10)	1712 (14.90)	11,489 (75.08)	
BMI (kg/m^2^)				
< 18.5	86 (30.82)	193 (69.18)	279 (0.49)	< 0.0001
18.5–< 30	41,624 (74.05)	14,585 (25.95)	56,209 (98.31)	
25–< 30	62 (30.85)	139 (69.15)	201 (0.35)	
30 +	290 (59.92)	194 (40.08)	484 (0.85)	
WHO				
I	8294 (69.28)	3678 (30.72)	11,972 (26.82)	< 0.0001
II	5370 (73.57)	1929 (26.43)	7299 (16.35)	
III	15,544 (75.65)	5004 (24.35)	20,548 (46.04)	
IV	3460 (71.90)	1352 (28.10)	4812 (10.78)	
Drug type				
First-line	40,981 (74.21)	14,240 (25.79)	55,221 (97.89)	0.3707
Second-line	868 (73.06)	320 (26.94)	1188 (2.11)	
ART duration				
≤ 1	10,488 (79.10)	2771 (20.90)	13,259 (23.19)	< 0.0001
> 1	31,574 (55.23)	12,340 (21.58)	43,914 (76.81)	
Adherence				
Good	40,000 (75.86)	12,731 (24.14)	52,731 (97.03)	< 0.0001
Poor	721 (44.62)	895 (55.38)	1616 (2.97)	
DTG-based regimen				
No	2087 (16.17)	10,819 (83.83))	12,906 (22.57)	< 0.0001
Yes	39,975 (90.30)	4292 (9.70)	44,267 (77.43)	
TB history				
No	42,015 (73.73)	14,971 (26.27)	56,986 (99.67)	< 0.0001
Yes	47 (25.13)	140 (74.87)	187 (0.33)	

### Socio-demographic factors and their association with LTFU

As depicted in Table 1, a slightly greater proportion of males (26.79%) than females (26.27%) were LTFU. Also, the age group of 15–24 years (33.64%) accounted for the highest proportion of LTFU, followed by the age group of 25–34 years (32.16%), respectively, compared to other age groups. Furthermore, pregnant women (37.14%) accounted for a significant proportion of LTFU compared to non-pregnant. A high proportion of patients from Kilindi (34.68%), Handeni (30.02), and, Muheza (29.96%) districts accounted for LTFU compared to the rest of the districts. In addition, single participants (29.67%) accounted for the highest proportion of LTFU compared to their counterparts.

### Clinical characteristics and its association with LTFU

Table 2 describes clinical factors and their association with LTFU. A slightly greater proportion (26.94%) of participants on the second-line regimen (drug type) were LTFU compared to those on first-line treatment (25.75%). Results showed that PLHIV on ART for more than 1 year were more likely (21.58%) to be LTFU than those on treatment for less than one year, but the difference was statistically significant. Of the 2,924 (19.35%) participants who did the viral load test, 14.90% who were virally suppressed were LTFU, compared to 31.78% who were virally non-suppressed. A greater proportion of participants (69.18%), and 69.15%, with BMI (kg/m^2^) < 18 and 25–< 30, respectively, were LTFU than those with a BMI between 18.5 – < 25 and 30 +. In addition, more participants in WHO stage I (30.72%) were LTFU than WHO stage II, III, and IV.

### Factors independently associated with LTFU among adults PLHIV in ART

We used a univariate logistic regression model to ascertain the independent variables contributing to LTFU. We included the variables in univariate analysis with a p < 0.05 in the multivariate logistic regression model (Table 3). Age between 15 and 24 (HR: 1.85, 95% CI 1.66–2.07) had about a two times higher risk of LTFU than other age groups, p < 0.0001, whereas males (HR: 2.00, 95% CI 1.51–2.62) have about twice the risk of being LTFU compared to females, p < 0.001. Divorced participants (HR: 1.35, 95% CI 1.24–1.48) were more likely to be LTFU than their counterparts. PLHIV on adult second-line drug type (HR: 1.13, 95% CI 1.09–1.18) were more likely to be LTFU than those in adult first-line drug type, p < 0.0001. Participants with poor drug adherence (HR: 1.50, 95% CI 1.23–1.75) had a 1.5 times higher risk of LTFU than those with good drug adherence, p < 0.0001. PLHIV with unsuppressed viral load (HR: 2.15, 95% CI 2.02–2.29) has about twice the risk of being LTFU compared to their suppressed counterparts. PLHIV who were not on DTG-related drugs (HR: 7.51, 95% CI 5.88–10.79) have about seven times increased risk of being to LTFU than those on DTG-related drugs, p < 0.0001. Additionally, PLHIV with WHO stage III and IV (HR: 2.51, 95% CI 2.32–2.72) had a higher risk of LTFU than their counterparts, p < 0.0001.

**Table 3:** Factors independently associated with LTFU among adult PLHIV on ART in the Tanga region

Study population characteristics	Univariate HR	p-value	Multivariate HR	p-value
	95% CI		95% CI	
Age group, years				
15–24	1.51 (1.47–1.55)		1.85 (1.66–2.07)	< 0.0001
25–34	1.22 (1.20–1.25)	< 0.0001	1.38 (1.28–1.48)	0.0011
35–44	Reference		Reference	
45–54	0.93 (0.91–0.95)		1.03 (0.96–1.11)	< 0.0001
55 +	1.04 (1.02–1.06)		1.20 (1.11–1.32)	0.1352
Sex				
Female	Reference	< 0.0001	Reference	
Male	0.94 (0.93–0.96)		2.00 (1.51–2.62)	< .0001
District				
Handeni	1.18 (1.15–1.20)		0.92 (0.83–1.02)	0.5184
Kilindi	1.38 (1.33–1.43)		0.58 (0.48–0.70)	< 0.0001
Korogwe	1.29 (1.26–1.32)	< 0.0001	0.85 (0.76–0.94)	0.00074
Lushoto	1.11 (1.08–1.14)		1.38 (1.25–1.53)	< 0.0001
Mkinga	1.14 (1.02–1.18)		0.41 (0.36–0.46)	< 0.0001
Muheza	2.49 (2.44–2.54)		1.22 (1.12–1.33)	< 0.0001
Pangani	1.22 (1.18–1.26)		2.13 (1.93–2.35)	< 0.0001
Tanga	Reference		references	
Pregnant status				
No	Reference		Reference	
Yes	1.10 (1.05–1.16)	< 0.0001	0.73 (0.63–0.85)	< 0.0001
Marital status				
Cohabiting	0.95 (0.90–1.01)		0.42 (0.35–0.52)	< 0.0001
Divorced	1.01 (0.99–1.04)	< 0.0001	1.35 (1.24–1.48)	< 0.0001
Married	Reference		Reference	
Single	1.31 (1.29–1.34)		1.25 (1.17–1.33)	< 0.0001
Widow	1.07 (1.04–1.10)		0.86 (0.76–0.96)	0.2
Viral load suppression				
Suppressed	Reference	< 0.0001	Reference	< 0.0001
Non-suppressed	2.30 (2.24–2.37)		2.15 (2.02–2.29)	
BMI (kg/m^2^)				
< 18.5	1.95 (1.84–2.07)	< 0.0001	1.75 (1.30–2.34)	0.0472
18.5–< 25	Reference		Reference	
25–30	1.57 (1.48–1.67)		1.46 (1.10–1.95)	0.5589
30 +	1.60 (1.50–1.68)		1.34 (0.93–1.93)	0.9229
WHO clinical stage				
I	Reference	< 0.0001	Reference	
II	0.01 (0.88–0.92)		1.67 (1.52–1.84)	0.1226
III	1.08 (1.06–1.09)		2.51 (2.32–2.72)	< 0.0001
IV	1.18 (1.15–1.21)		2.24 (2.02–2.49)	< 0.0001
Drug type				
First-line	Reference	< 0.0001	Reference	< 0.0001
Second-line	1.13 (1.09–1.18)		1.19 (0.84–1.69)	
Adherence				
Good	Reference	< 0.0001	Reference	
Poor	1.75 (1.69–1.82)		1.50 (1.23–1.75)	< 0.0001
ART duration				
≤ 1	1.10 (1.08–1.12)	< 0.0001	0.72 (0.65–0.81)	< 0.0001
> 1	Reference		Reference	
TB history				
No	Reference	< 0.0001	Reference	
Yes	1.92 (1.79–2.05)		1.19 (0.84–1.69)	0.3187
DTG-related drug				
No	5.80 (4.98–6.64)	< 0.0001	7.51 (5.88–10.79)	< 0.0001
Yes	Reference		Reference	

Cohabiting (HR: 0.42, 95% CI 0.35–0.52), pregnant women (HR: 0.73, 95%CI 0.63–0.85), and ART duration of less than 1 year (HR: 0.72, 95% CI 0.65–0.81) were the factors that reduced the likelihood of LTFU. While variables on tuberculosis history, WHO stage II, BMI, being widowed, and age above 55 + were statistically significant in the univariate analysis but lost their statistical significance level in the multivariate analysis, i.e., they were not the factors that were independently associated with LTFU.

## Discussion

Effective use of antiretroviral drugs reduces the risk of HIV transmission and HIV-related morbidity, improves the quality of life, and reduces the risk of drug resistance. However, ART care and treatment interruption affect drug effectiveness [2]. This retrospective study described the rate and predictors of loss to follow-up (LTFU) in antiretroviral therapy experienced adult patients in the Tanga region. First, the overall rate of LTFU among adults PLHIV on ART was 26.43% observed in 2 years. Second, the study found that young age (15–24 years), male sex, use of the second-line drug, poor drug adherence, unsuppressed viral load, not using DTG related-drugs, and advanced HIV disease WHO stage III & IV to be the factors independently associated with LTFU. Third, the study found that cohabiting, having an ART duration of less than one year, and being pregnant have a reduced risk of LTFU. Fourth, having a tuberculosis history, WHO stage I and II, BMI of 25–30, age above 55 years, and patients reported to be widowed were not the factors independently associated with LTFU.

The large sample size and long duration of observation of the cohorts on ART allow generalizability of the study findings for the people living with HIV on ART in the Tanga region. However, the current study missed out on assessing important variables, like CD4 count, education level, and social support status, that are also reported to predict LTFU significantly.

The prevalence of LTFU in adults PLHIV in the current study was similar to the findings of studies conducted in Nigeria and Gabon, where the cumulative rate of LTFU at 13- and 10-years review in Nigeria was 30.6% and 32% [19, 20], while in Gabon in a 2 years review was 34.1% [21]. The study findings are in contrast to the 13.45% LTFU in a retrospective study in Northwest Ethiopia [22], 3% LTFU in a study in France [23], 12.75% LTFU in a retrospective study in Kampala-Uganda [24], 12.8% LTFU in a cohort study in South Africa [25], while a study done in Guinea and Kenya reported a prevalence of 57.61% and 54% LTFU from a 7 years follow-up study [26, 27]. The observed variation in LTFU prevalence might be due to heterogeneity of the population studied and disparity in strategies used to track patients. Advanced HIV disease, transportation, opting to use herbal medicine, work responsibilities, not disclosing HIV status, religious beliefs, and financial challenges are the probable reasons to interrupt care and treatment among our study population [9, 28].

The current study had more females than males, but males were two times more likely to be LTFU. The findings are consistent with studies [5, 8, 19, 26] where the male gender was independently associated with LTFU in HIV/AIDS programs. The possible reason men are predisposed to LTFU is that men often present for ART care and treatment at a later stage compared to women, with an increased risk of severe illness and death [29]. Therefore, the increased risk of LTFU observed amongst men in most studies, including the current research, signifies a need for specific gender-based intervention to reduce LTFU.

Young people aged 15–24 were at higher risk of LTFU than those aged 25 and above. This aligns with findings from previous studies [5, 30–32], but the finding contrasts with results from a study [19, 21, 24] that found increasing the age of people living with HIV was associated with LTFU. The reasons why the young generation is at high risk for LTFU are lack of status disclosure within the family, school pressure, having unstructured lives, fear of stigma and discrimination, and inadequate support from guardians and parents [33–35]. Therefore, developing strategies for retaining youths in ART care and treatment is critical to improving long-term HIV outcomes for youths.

WHO stages III and IV were predictors of loss to follow-up from ART care and treatment. The risk was 2.51 times higher compared to WHO clinical stage I. The finding is consistent with studies in Ethiopia, Uganda, South Africa, and Malawi [24, 29, 36, 37]. The significant prevalence of immunological impairment in HIV-positive patients with WHO clinical stage IV may explain the high risk of infections, and they are bedridden most of the time. As a result, they might not meet their care and therapy tasks. In addition, these patients have an increased risk of death because of the significant immunosuppression. Therefore, it is possible that they died and got reported to the ART clinic again, indicating poor documentation for the event. Therefore, designing and implementing strategies to improve retention rates among patients with advanced HIV clinical stages is crucial. Despite focusing on only offering ART, program implementers may also be concerned about knowing the health status of the patients while at home by visiting them before coming for the next appointment to refill the drug.

Antiretroviral therapy drug adherence was a statistically significant predictor of LTFU. People living with HIV with poor ART drug adherence had a 1.5 times higher risk than those with good ART drug adherence. This finding is consistent with the study conducted in Gonda, sub-Saharan Africa, and low and middle-income countries meta and systematic analysis [31, 38, 39]. As said earlier in this study, reasons like interference with religious belief, use of herbal medicine as an alternative drug, fear of discrimination, drug side effects, and financial crisis might have contributed to poor drug adherence. So, healthcare providers can find out the possible reasons for poor adherence from patients and propose strategies to program implementers to improve drug adherence.

Our study found that adults in second-line ART regimens had a higher risk of LTFU than those in the first-line. Our finding is similar to the studies conducted in Nigeria [19, 20] and Uganda [24], which reported a high risk of LTFU in treatment among patients on a second-line regimen. However, the finding is inconsistent with studies [31, 36] that found patients on 1st line regimens to have an increased risk of LTFU. The observed variation might be due to the side effects caused by each regimen and the person's drug tolerance level. Maybe most of the study participants in the current study could not tolerate the side effects of the second-line regimen. Therefore, continuing to closely monitor patients in different drug regimens and their tolerance levels and taking appropriate action is paramount to ensure no LTFU due to drug intolerance.

Additionally, our study found that patients who were not on a DTG-based regimen had about seven times increased risk of being LTFU than those on a DTG-based regimen, p < 0.001. To the best of our review, limited studies looked at the association between LTFU and DGT-based regimen use to help discuss the present study's findings. Further study that looks at the association of LTFU and DTG use is warranted to inform policymakers to decide.

Lastly, findings show that patients with TB had a higher risk of LTFU than those without TB. The results align with the studies [8, 40, 41] that found TB patients on ART have more risk of LTFU than their counterparts. HIV patients are already immunocompromised, and having additional TB disease would further weaken the immune system. Without further and appropriate treatment with close follow-up, diseases can progress from sickness to death and contribute to many LTFUs if not correctly documented.

## Conclusion

Advanced HIV WHO clinical stage III and IV, male gender, second-line regimen, non-virally suppression, non-use of DTG-based regimen, young age, and poor drug adherence were independently associated with LTFU. Therefore, to reduce LTFU, program implementers might find out the strategies targeting the presented risk factors in the present study to help improve the retention of ART to reach the HIV/AIDS eradication plan of 2030.

## Data Availability

The datasets generated and/or analyzed during the current study are not publicly available due to sensitive information about the participants. Also, the participants did not provide their approval for the sharing of their information. However, data are available from the Directorate of National Aids Control Program (contact via nacp@afya.go.tz) on reasonable request.

## Abbreviations

ART: Antiretroviral therapy
BMI: Body mass index
CI: Confidence interval
CTC2: Care and treatment form number two
DTG: Dolutegravir
LTFU: Loss to follow-up
PLHIV: People living with human immunodeficiency virus
HIV: Human immunodeficiency virus
WHO: World Health Organization
